# Integrating proteomics and network pharmacology to explore the relevant mechanism of Huangkui capsule in the treatment of chronic glomerulonephritis

**DOI:** 10.3389/fphar.2025.1560420

**Published:** 2025-04-28

**Authors:** Chang Qing Wen, Jia Zou, Jia Xuan Li, Fu Jiang Wang, Hai Tao Ge

**Affiliations:** ^1^ School of Basic Medicine and Clinical Pharmacy, China Pharmaceutical University, Nanjing, Jiangsu, China; ^2^ Jiangsu Suzhong Pharmaceutical R&D Institute Co., Ltd., Nanjing, Jiangsu, China

**Keywords:** Huangkui capsule, proteomics, network pharmacology, chronic glomerulonephritis, mechanism

## Abstract

**Introduction:**

Chronic glomerulonephritis (CGN) is a common glomerular disease with multifactorial pathogenesis. Huangkui capsule (HKC), a traditional Chinese herbal formulation, demonstrates therapeutic potential in CGN; however, its molecular mechanisms remain insufficiently characterized. This study aimed to clarify the therapeutic mechanisms of HKC in CGN by integrating proteomic analysis with network pharmacology.

**Methods:**

We employed liquid chromatography–mass spectrometry (LC-MS) to identify the active components of HKC. A CGN rat model was established and treated with HKC. Renal function parameters and serum inflammatory cytokines were assessed. Histopathological alterations and IgG deposition in kidney tissues were examined using hematoxylin–eosin (HE) staining and immunofluorescence, respectively. Proteomic profiling of renal tissue was conducted, and network pharmacology analysis was applied to identify potential therapeutic targets of HKC.

**Results:**

A total of 39 active compounds were identified in HKC. HKC administration significantly improved renal function and mitigated glomerular injury in CGN rats. Proteomic analysis revealed 2,079 differentially expressed proteins, predominantly associated with oxidoreductase activity. Network pharmacology identified 462 targets related to HKC and 1,835 targets associated with CGN, with 13 overlapping targets, including STAT3, PIK3R1, AKT1, HIF-1α, and VEGF, which were downregulated following HKC treatment.

**Conclusion:**

HKC exerts renoprotective effects in CGN by regulating multiple signaling pathways, notably HIF-1, VEGF, PI3K-Akt, MAPK, and PPAR. Through attenuation of inflammatory and oxidative responses, HKC alleviates renal pathological damage and supports kidney function, offering mechanistic insight into its multi-target therapeutic potential.

## 1 Introduction

**FIGURE 1:**
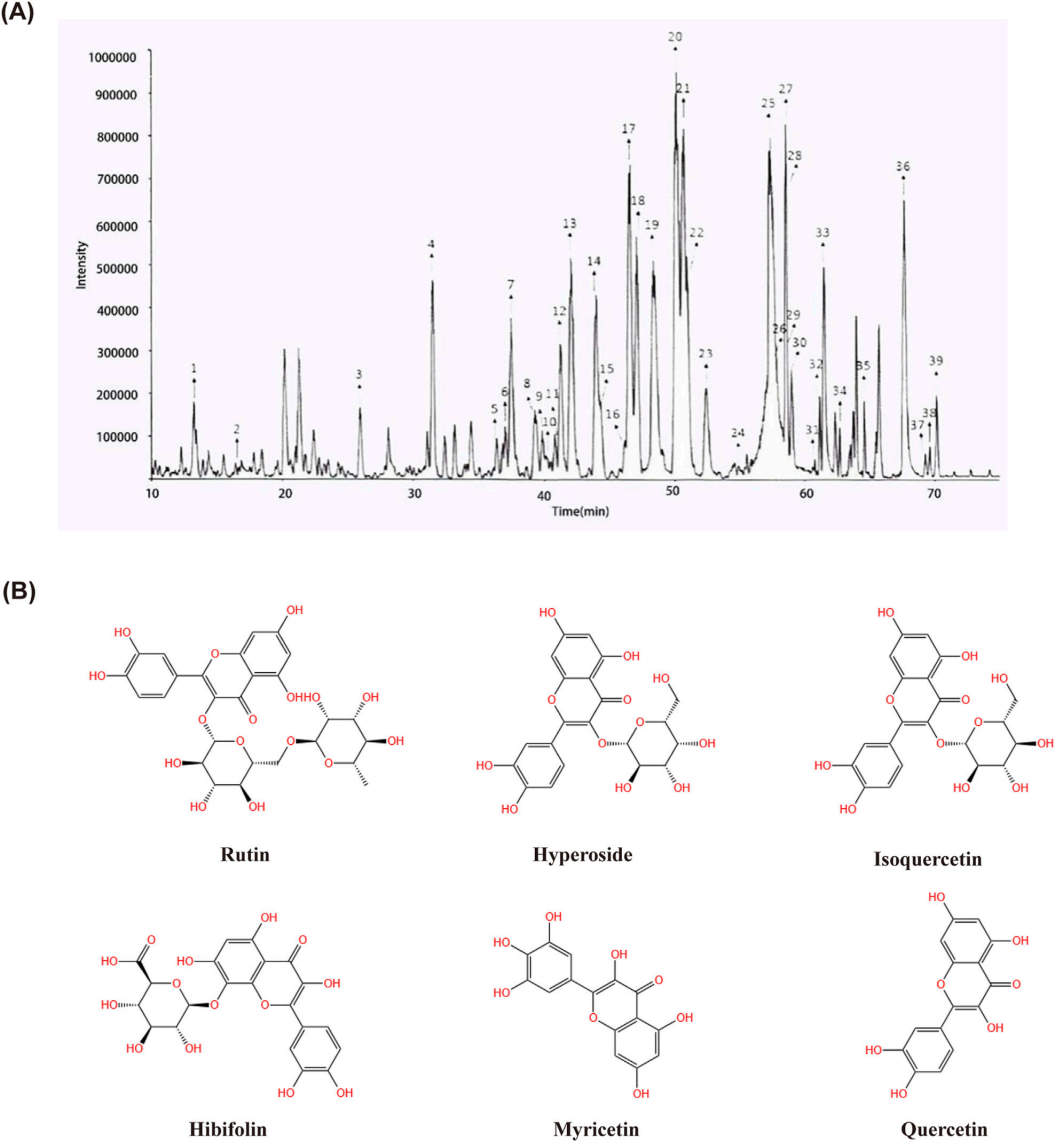
Determination of 39 compounds in Huangkui capsule by LC-MS/MS.18. Rutin; 20. Hyperoside; 21. Isoquercetin.; 25. Hibifolin; 33. Myricetin; 36. Quercetin. **(A)** Structural formulae of Rutin, Hyperoside, Isoquercetin, Hibifolin, Myricetin and Quercetin **(B)**.

CGN is a common immune-mediated glomerular disorder characterized by proteinuria, hematuria, hypertension, and edema, often leading to renal insufficiency and, ultimately, end-stage renal disease (ESRD) (Chebotareva et al., 2015; Satirapoj et al., 2015). The global burden of CGN is rising, with mortality increasing annually from 2015 to 2020, reaching 52,260 deaths in 2020 (Foti et al., 2021). In China alone, chronic kidney disease (CKD) affects approximately 132.3 million individuals, contributing nearly one-fifth of the global burden (Kriz et al., 1998). Research suggests that cytokine- and immunokine-mediated microcirculatory disturbances, cellular proliferation, and immune complex deposition are key pathological mechanisms (Ahmad and Bomback, 2020). *Abelmoschus manihot* (L.) Medic (AM), a traditional Chinese medicine, has been used in China for centuries. HKC, a single-herb traditional Chinese medicine preparation derived from AM corolla extract. It was approved by the China Food and Drug Administration (CFDA) in 1999 (Z19990040).The primary active components of AM are flavonoids, including rutin, hyperoside, hibifolin, isoquercitrin, myricetin, quercetin, and quercetin-3-O-robinobioside (Guo et al., 2015; Li et al., 2021). The chemical spectrum and structural formulas of these compounds are shown in Figure 1. *In vivo*, these flavonoids can be metabolized into sulfate-glucuronide conjugates, which are considered key contributors to the nephroprotective effects of HKC (Guo et al., 2016). Multiple multicenter randomized controlled trials (RCTs) have confirmed that HKC improves renal function in patients with primary glomerular diseases, IgA nephropathy, and diabetic nephropathy (DN) (Li et al., 2017; Li et al., 2020; Zhao et al., 2022). Compared with conventional medications such as angiotensin-converting enzyme (Regev et al., 2022) inhibitors and angiotensin receptor blockers (ARBs), HKC demonstrates distinct advantages in treating chronic kidney disease (CKD), as it improves renal function and reduces proteinuria without adverse effects on blood pressure. Both preclinical and clinical studies consistently indicate that HKC alleviates renal injury, reduces urinary protein excretion, and improves renal function (Cai et al., 2017). The key active ingredients, including quercetin, myricetin, rutin, and hyperoside, exert therapeutic effects via multitarget mechanisms, such as anti-inflammatory, antioxidant, and immunomodulatory pathways (Wan et al., 2022). Therefore, with its remarkable renoprotective effects, HKC has gradually become the clinical treatment of choice for CGN (Pei and Li, 2021). However, studies on its direct targets and signaling pathways are still limited, and the specific mechanisms remain to be elucidated (Liu et al., 2012).

This study will combine network pharmacology and proteomics to investigate the effectiveness and potential molecular mechanism of CGN treatment with HKC, which will provide new perspectives and scientific references for further revealing its potential pharmacological effects, as well as provide theoretical basis for its clinical application and modernization research.

## 2 Materials and methods

### 2.1 Reagents

HKC (Batch number: 22041603), produced by Suzhong Pharmaceutical Group Co., Ltd.; Losartan Potassium tablets, produced by Yangtze River Pharmaceutical Group (Batch number: 21060204). All ELISA kits are purchased from enzyme-linked immunosorbent assay (Jiangsu Meimian industrial Co., Ltd, Jiangsu, China).

### 2.2 Material basis of HKC

Extract of HKC 114 mg, dissolved in 50% methanol, sonicated for 10 min, centrifuged at 10,000 rpm for 10 min. Chromatographic conditions: The chromatographic column is Waters Xbridge Shield RP18 (4.6 × 250 mm, 5 µm), 0.05% formic acid aqueous solution (V/V) as mobile phase A, 0.05% formic acid acetonitrile solution as mobile phase B, gradient elution (0–10 min, 5%–10% B; 10–30 min, 10%–18% B; 30–40 min, 18%–20% B; 40–50 min, 20%–24% B; 50–60 min, 24%–40% B; 60–80 min, 40%–60% B; 80–90 min, 60%–100% B); The flow rate is 0.7 mL/min; The injection volume is 5 μL.

LCQ Deca Xp plus mass spectrometry conditions: electric spray ion source (ESI), negative ion scanning mode, scanning range: m/z 100–1,400, sheath gas and auxiliary gas are high purity nitrogen, sheath gas flow rate: 60 arb, auxiliary gas flow rate: 20arb; The collision gas is high-purity helium, source voltage: 3 kV, capillary temperature: 350°C, capillary voltage: −15 V.

### 2.3 Modeling

Forty male Sprague-Dawley rats of SPF grade, provided by Nantong University. Animal permit number: SCXK (Su) 2019-0001. The animals were housed in the SPF-grade animal laboratory of Jiangsu Suzhong Pharmaceutical Group Biopharmaceutical Co., Ltd. The experimental animal permit number is SYXK (Su) 2017-0059. The rats were acclimated to the environment for 1 week prior to the start of the experiment, provided with regular pellet feed, and had free access to food and water. The temperature in the room was maintained at 20°C–24°C, with a humidity of approximately 45%–65%, and a 12-h light-dark cycle. All animal studies were approved by the Animal Ethics Committee of Jiangsu Suzhong Biopharmaceutical Co., Ltd. (Approval number: SZSW2022010602).

**FIGURE 2:**
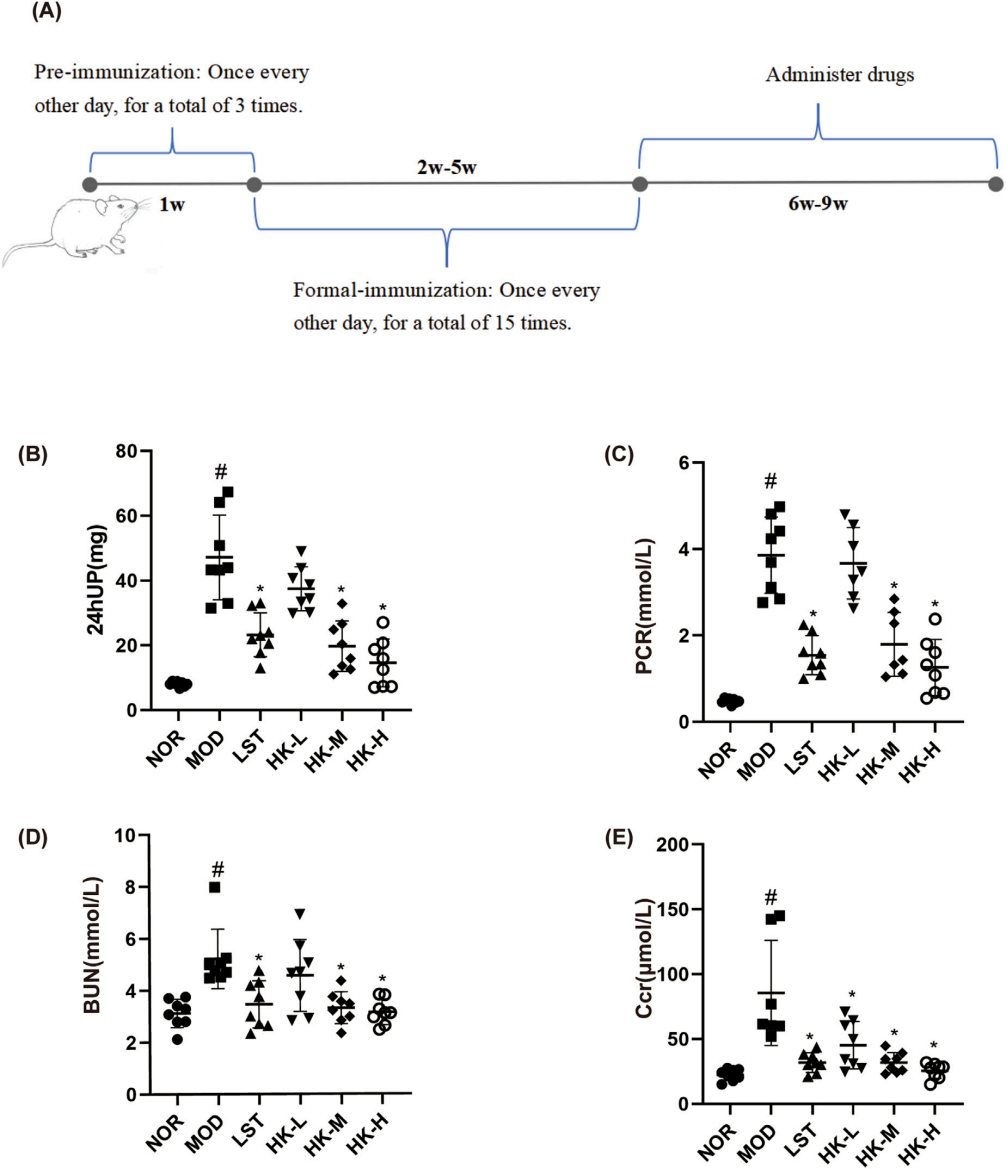
Schematic diagram of CGN rat model establishment **(A)**. 24hUP, PCR, BUN, and Scr levels in each group of rats (n = 8). Compared with the NOR group, the 24-h urine protein content **(B)**, PCR **(C)**, BUN **(D)** and Scr **(E)** values in the model group were significantly higher (P < 0.05); compared with the MOD group, the 24-h urine protein content, PCR, Scr and BUN values in the LST, HK-M and HK-H groups were significantly lower (P < 0.05). ^#^p<0.05 vs NOR group; *p<0.05 vs MOD group.

After 1 week of adaptive feeding, 40 SD rats were used to establish the CGN model. As shown in Figure 2A, pre-immunization: Firstly, dissolve 1 mg of C-BSA in 0.5 mL of 0.9% NaCl solution. Then, add 0.5 mL of Freund’s incomplete adjuvant to the solution. After complete emulsification, inject 1 mL of the emulsion into the rats (injection sites: both forelimb axillae and bilateral inguinal regions, injection route: subcutaneous, injection frequency: once every other day, 3 times per week) for 1 week of pre-immunization. (Tian et al., 2019). Formal immunization: each rat was injected via the tail vein every other day with C-BSA (16 mg/kg), 3 week, for a total of 15 injections. After model induction, the rats’ urine protein content was measured for 24 h, and if it exceeded 20 mg, the model was considered successful. Normal controls and received an equivalent volume of saline in the same manner.

The successfully modeled rats were divided into the following groups: model group (MOD), losartan potassium group (LST), HKC low-dose group (HK-L), HKC medium-dose group (HK-M), and HKC high-dose group (HK-H), with eight rats in each group. Rats in the HK-L, HK-M, and HK-H groups were respectively given interventions with 270 mg/kg, 540 mg/kg, and 1,080 mg/kg of AM extract (one HKC contains 0.4 g of AM extract powder, and the medium dose is equivalent to the clinical effective dose). The LST group was intervened with a dose equivalent to the clinical effective dose of 4.5 mg/kg. The medications were prepared using purified water. The normal group and the model group received equal volumes of purified water via oral gavage, once a day, four consecutive weeks.

### 2.4 Biochemical index analysis

Following a 4-week treatment with HKC, metabolic cages were utilized to conduct 24-h urine collections for each group of rats. The 24-h urine protein content was measured using the kit. The urinary creatinine and serum creatinine (Scr) concentrations were measured using a microplate assay. The blood urea nitrogen (BUN) concentration was measured using the urease method. Detection of PCR using kits. PCR stands for the ratio of urinary protein to creatinine, an important indicator used to evaluate the amount of protein excreted in the urine, and an increase in urinary protein represents kidney damage.

To collect blood samples, the rats were administered anesthesia with 10% chloral hydrate (0.3 mL/100 g) followed by the extraction of blood from the abdominal aorta using a blood collection tube and needle. The activities of superoxide dismutase (SOD) and malondialdehyde (MDA) levels related to oxidative stress were measured according to the steps described in the kit. The kidney on the right side underwent cryopreservation using liquid nitrogen and was subsequently enveloped in aluminum foil. This facilitated its storage at a temperature of −80°C, enabling proteomics analysis. Conversely, the kidney on the left side was subjected to fixation in a 10% formalin solution to facilitate subsequent histopathological observation.

### 2.5 ELISA

The concentrations of IL-6 and TNF-α in renal tissue homogenates were assessed using the enzyme-linked immunosorbent assay (ELISA) technique. Initially, 100 µL of immobilized IL-6 and TNF-α antibodies were introduced into the ELISA plate and allowed to incubate overnight at ambient temperature. After washing the plate and blocking it for 1 h, 100 µL of rat renal tissue homogenate was added to each well. Each sample was replicated three times. The plate was then incubated at room temperature for 3 h, followed by washing. After incubating with the corresponding enzyme-linked secondary antibodies for 30 min and washing again, the substrate solution was added. The levels of IL-6 and TNF-α in homogenized renal tissue were quantified using the enzyme-linked immunosorbent assay (ELISA) method. Initially, 100 µL of IL-6 and TNF-α antibodies were added to the ELISA plate and incubated overnight at room temperature. Following plate washing and a subsequent 1-h blocking step, 100 µL of rat renal tissue homogenate was added to each individual well. Each sample was replicated three times. The plate was then incubated at room temperature for 3 h, followed by another washing step. Following a 30-min incubation with the corresponding secondary antibodies linked to enzymes and subsequent washing, the substrate solution was added. The absorbance was read at 405 nm wavelength to calculate the concentrations of IL-6 and TNF-α.

### 2.6 Histopathological and immunofluorescence staining of the kidney

The renal tissues from each group of rats were fixed in a 10% formalin solution for 24 h. Afterward, they were washed with water for 20 min, dehydrated using an alcohol gradient, cleared with xylene, embedded in paraffin, and sliced into 5-µm sections. Subsequently, the tissue sections were subjected to hematoxylin and eosin (HE) staining to facilitate microscopic examination and enable the observation of fundamental pathological alterations in the renal tissues of each experimental group. And for Immunofluorescence, the kidney sections were processed using standard methods. The secondary antibodies were coupled at room temperature and then incubated with DAPI. And finally mounted with an anti-fluorescence quenching agent for slide sealing and scanning. The relative fluorescence intensity was analyzed using ImageJ software.

### 2.7 Drug and disease target prediction

The HKC target proteins were predicted using the Swiss target Prediction (https://www.swisstargetprediction.ch/) databases. The CGN disease targets were predicted using the GeneCards (www.genecards.org) databases. The CGN disease and HKC targets were predicted using the GeneCards (www.genecards.org) databases.

### 2.8 Quantitative proteomics

#### 2.8.1 Sample production

Sample production is entrusted to the GeneDenovo Co. (Guangzhou, Guangdong, China).

#### 2.8.2 Differential protein selection

The results were visualized using volcano plots and hierarchical clustering analysis heatmaps. Furthermore, proteins that showed large fold change differences after intervention with NOR, MOD, and HK groups and were potentially related to kidney diseases were presented in tabular format.

#### 2.8.3 Bioinformatics analysis of differential proteins

(1) GO Enrichment Analysis of Differential Proteins

Differential proteins were imported into the Metascape database to obtain GO annotations and enrichment information, which provided insights into the biological functions in which differential proteins are involved and the number of proteins enriched in those functions, using a filtering condition of p < 0.05.

(2) KEGG Enrichment Analysis of Differential Proteins

Selected differential proteins were imported into the Metascape database to obtain KEGG enrichment information, which revealed the signaling pathways and processes in which differential proteins are involved, as well as the number and types of proteins enriched in those pathways.

(3) PPI Network Construction and Core Target Selection of Differential Proteins

To investigate the relationships between targets, a protein-protein interaction (Tsao et al., 2021) network of differential proteins was constructed using the STRING database.

### 2.9 Statistical method

The statistical software GraphPad Prism 8.0 was used to analyze the experimental data. Data were expressed as mean ± standard deviation, a t-test was applied for comparisons between two groups, and a one-way analysis of variance (ANOVA) was applied for comparisons between multiple groups. A p-value of less than 0.05 (p < 0.05) was considered statistically significant, as determined by one-way ANOVA followed by Tukey’s *post hoc* test for multiple comparisons.

## 3 Results

### 3.1 Material basis of HKC

The chromatogram of HKC extract is shown in Figure 1. For components with reference standards, the chemical structure of the components is identified by comparing them with information such as retention time and mass spectrometry data of the reference standards; For components without reference standards, their molecular formula can be inferred based on the precise molecular weight obtained from high-resolution mass spectrometry, and their chemical structure can be inferred by combining information from multi-level mass spectrometry and data reported in literature. According to the above method, a total of 39 components have been identified or speculated.

### 3.2 HKC effects on renal function and pathological manifestations in CGN rats

The renal function test results showed that compared to the normal group, the model group of rats had significantly increased levels of 24-h urinary protein, PCR, Scr, and BUN (P < 0.05). In comparison to the model group, the valsartan potassium group, the middle dose of HKC group, and the high dose of HKC group exhibited significant reductions in 24-h urinary protein, PCR, Scr, and BUN levels (P < 0.05) (Figures 2A–D).

**FIGURE 3:**
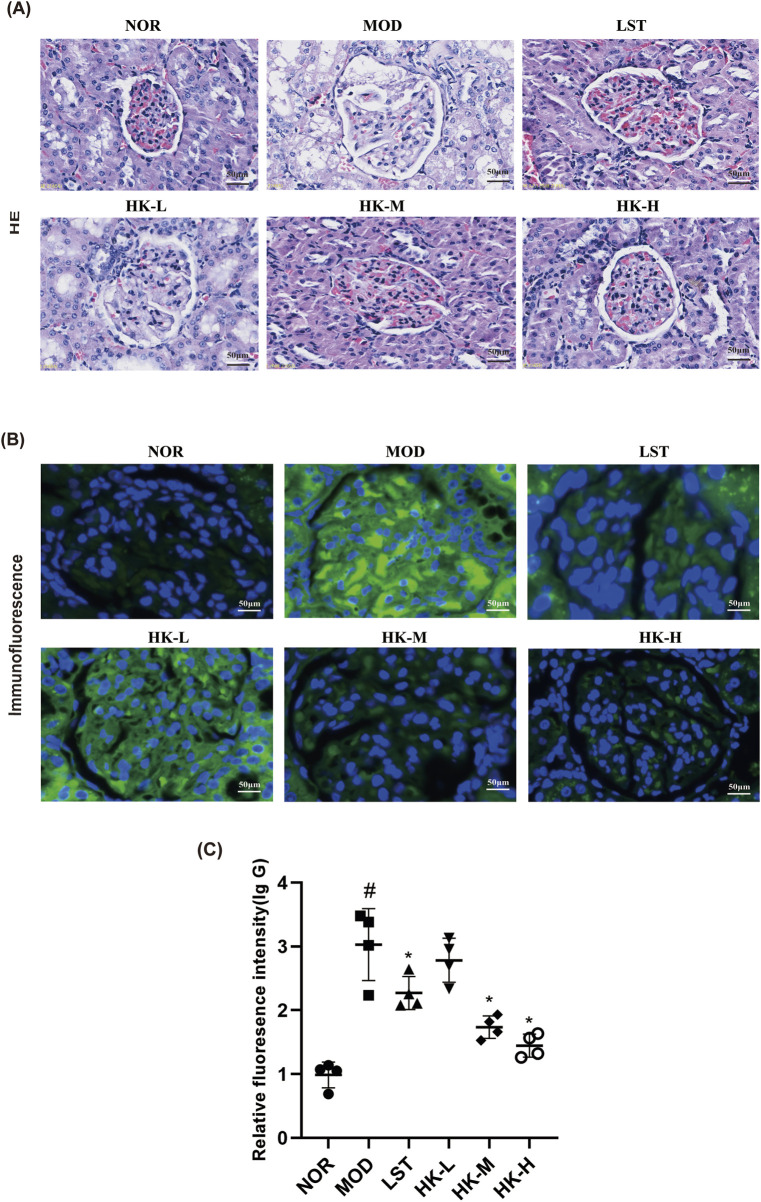
Statistical results of relative intensity of HE staining, immunofluorescence staining and IgG immunofluorescence (n = 8). **(A)** HE staining showed that the glomeruli of rats in the NOR group were structurally intact and the size of the capsule was normal, while the glomeruli of rats in the MOD group were enlarged in size and the basement membrane was thickened to different degrees. Compared with the MOD group, the increase in glomerular volume and the thickening of basement membrane of rats in the NOR group were reduced after treatment with Huangkui capsule and chlorosartan potassium. **(B, C)** At the same time, immunofluorescence results showed that there was almost no IgG deposition in the renal tissues of rats in the NOR group, while in the MOD group, IgG was obviously seen to be diffusely deposited in the glomerular mesangium or basement membrane, and the phenomenon of IgG deposition in the glomeruli of the rats in all groups was significantly reduced after treatment with Huangkui Capsule and Losartan Potassium. ^#^p < 0.05 vs. NOR group; *p < 0.05 vs. MOD group.

HE staining demonstrated that the renal glomerular structure of rats in the normal group was intact, and the size of the cysts was normal. In the model group of rats, the renal glomeruli exhibited increased volume and varying degrees of thickening of the basement membrane. Comparing with the model group, rats treated with HKC and valsartan potassium showed reduced enlargement of the renal glomeruli and decreased thickening of the basement membrane. Additionally, immunofluorescence results revealed minimal IgG deposition in the renal tissues of rats in the normal group, while the renal tissues of rats in the model group exhibited significant diffuse IgG deposition in the glomerular mesangium or basement membrane. After treatment with HKC and valsartan potassium, the deposition of IgG in the renal glomeruli of rats in each group was significantly reduced (Figure 3).

To further evaluate glomerular histomorphology, H&E-stained sections from each animal were selected, and glomerular interstitial area was semi-quantified using Olympus Image Viewer (version 3.1.1) software (Table 1). Furthermore, based on the Ehrenreich-Churg staging criteria for membranous nephropathy (Alsharhan and Beck, 2021; Stefan et al., 2023), the degree of basement membrane thickening was classified as mild, moderate, or severe (denoted by *, **, ***). IgG deposition was graded by immunofluorescence intensity as weakly positive, strongly positive, or multi-antibody strongly positive (denoted by *, **, ***). The results are summarized in Table 2.

**TABLE 1:** Glomerular interstitial area.

Group	Glomerular interstitial area (Mean) μm^2^	Statistical discrepancy
NOR	6,492 ± 372.5	P < 0.0001(NOR vs. MOD)
MOD	15,697 ± 164.8	
LST	7,346 ± 198.1	P < 0.0001 (LST vs. MOD)
HK-L	8,192 ± 245.8	P < 0.0001(HK-L vs. MOD)
HK-M	7,947 ± 183.0	P < 0.0001(HK-M vs. MOD)
HK-H	7,211 ± 181.3	P < 0.0001(HK-H vs. MOD)

**TABLE 2:** Glomerular pathology scale.

Groups	Thickening of the basement membrane	Deposition of immune complexes (IgG)	Comprehensive evaluation
Normal	N/A	N/A	N/A
Model	***	***	+++
Low-dose	**	**	++
Medium-dose	*	*	+
High-dose	*	*	+
Losartan	*	*	+

Scoring of basement membrane thickening: N/A: none, *: Mild thickening of the glomerular basement membrane (GBM), **: Moderate thickening of the GBM, ***: Marked thickening of the GBM., Scoring of IgG deposition: N/A: none, *: Weak positive for IgG deposition, **: Strong positive for IgG deposition, ***: Strong positive for multiple antibodies (“full-house” pattern). Comprehensive evaluation: Add up the number of * for basement membrane thickening and IgG deposition. +: *∼**, ++: ***∼****,++: *****∼******.

### 3.3 HKC effects on oxidative stress and inflammatory actors in CGN rats

**FIGURE 4:**
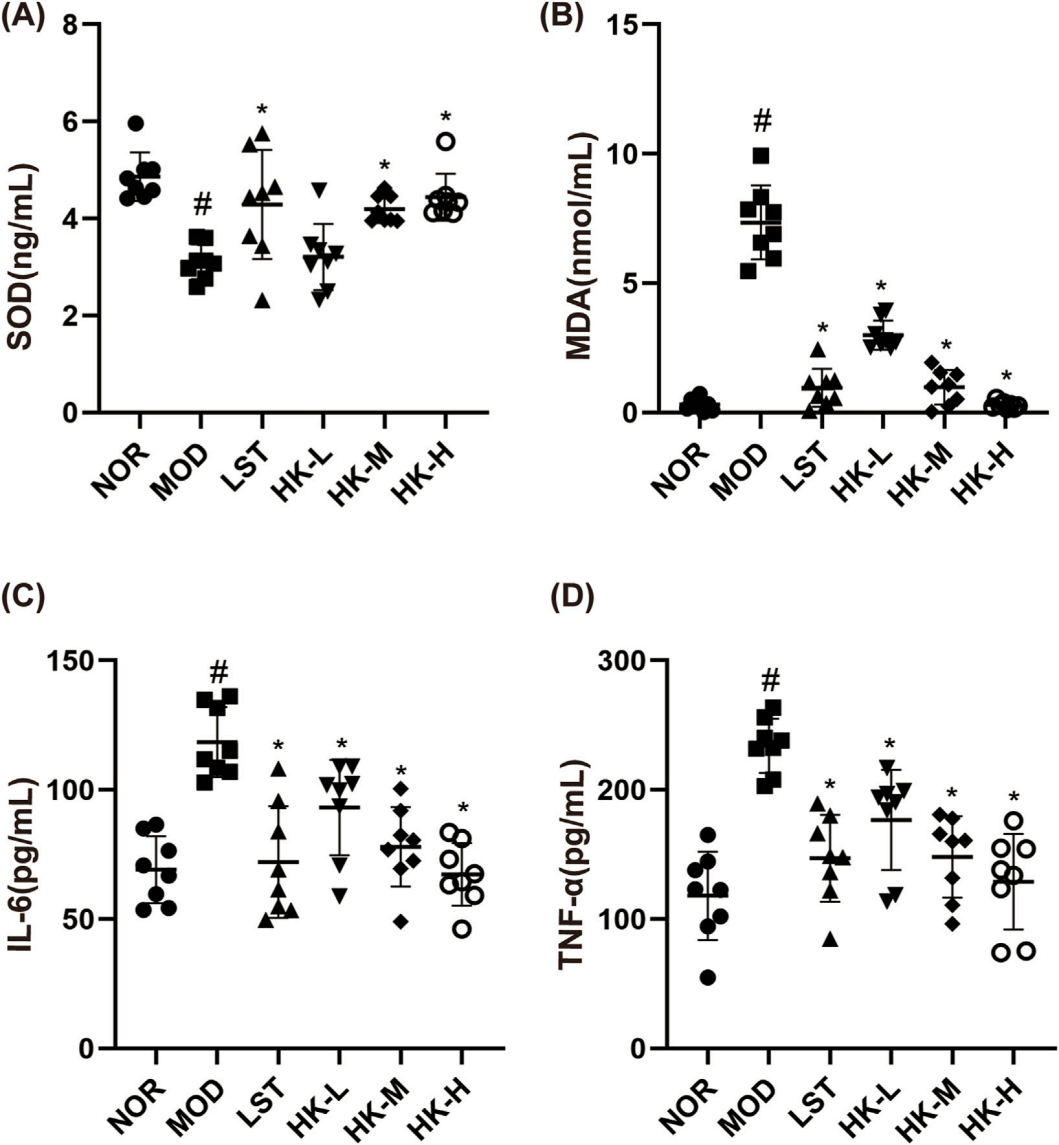
Results of SOD\MDA\IL-6 and TNF-α content in rat serum (n = 8). Compared with the NOR group, the serum TNF-α **(D)**, IL-6 **(C)** and MDA **(B)** levels in the MOD group were significantly higher (P < 0.05), and the SOD **(A)** activity was significantly lower (P < 0.05); and compared with the MOD group, the serum TNF-α **(D)**, IL-6 **(C)** and MDA **(B)** levels in the LST, HK-M and HK-H groups were significantly lower (P < 0.05), and the SOD **(C)** activity was significantly higher (P < 0.05). levels were significantly decreased (P < 0.05) and SOD **(C)** activity was significantly increased (P < 0.05). ^#^p < 0.05 vs. NOR group; *p < 0.05 vs. MOD group.

The effects of HKC on oxidative stress in CGN rats were further explored by measuring the SOD and MDA levels in the blood of rats in each group. The enzyme-linked immunosorbent assay (ELISA) was employed to assess the concentrations of pro-inflammatory cytokines, including IL-6 and TNF-α, in the renal tissue homogenates of rats from each experimental group. This analysis aimed to evaluate the impact of HKC on the inflammatory response. In comparison to the control group, the model group of rats exhibited notable elevations in the serum levels of TNF-α, IL-6, and MDA (P<0.05), accompanied by a significant reduction in SOD activity (P<0.05). The valsartan potassium group, the middle dosage of HKC group, and the high dose of HKC group demonstrated marked reductions in serum TNF-α, IL-6, and MDA concentrations (P<0.05) as well as a notable boost in SOD activity (P<0.05), in contrast to the experimental group (Figure 4).

### 3.4 Network pharmacology analysis

**FIGURE 5:**
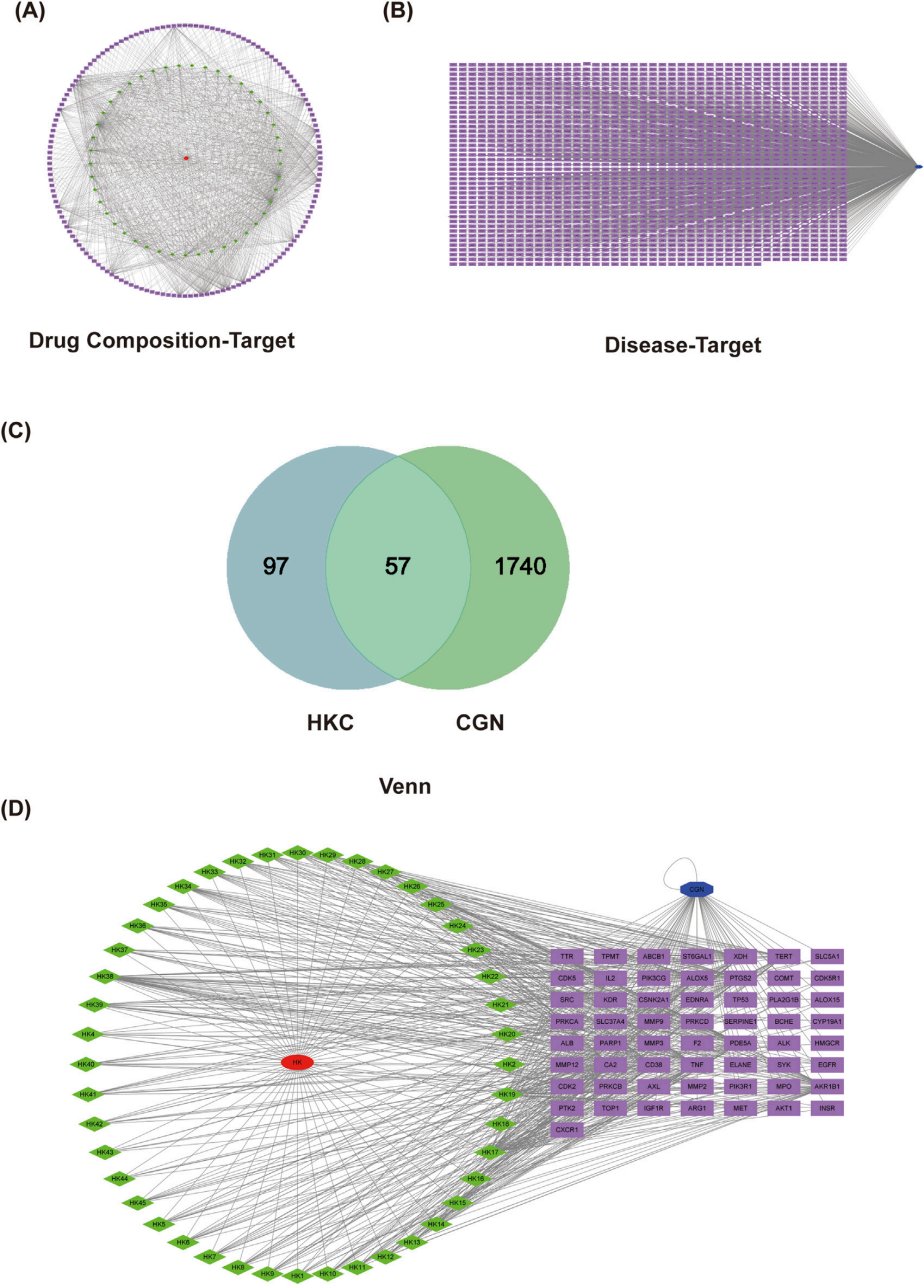
Network pharmacology target analysis. **(A)** Chronic glomerulonephritis target prediction and drug-disease target collection. **(B)** PPI network diagram of the overlapping targets.

To explore the impact of HKC on gene expression in the renal tissues of rats with CGN, a network pharmacological prediction approach was employed. This methodology aimed to unravel the molecular mechanism underlying the therapeutic effects of HKC in CGN treatment, following the guidelines provided in the Network Pharmacology Evaluation Method. We collected 39 HKC chemical compounds along with their corresponding structures for exploration of component, targets and diseases. Using the Swiss Target prediction database, we predicted 462 potential targets for HKC. Then, referring to the GeneCards database, we found 1,835 targets associated with CGN. By taking the intersection of the two sets, we obtained 147 common targets. A PPI (Protein-Protein Interaction) network diagram was constructed for these core intersecting targets, which included key core genes such as TNF (Tumor Necrosis Factor), ALB (Albumin), AKT1 (Protein Kinase B), STAT3 (Signal Transducer and Activator of Transcription 3), CASP3 (Signal Transducer and Activator of Transcription 3), EGFR (Epidermal Growth Factor Receptor), and MMP9 (Matrix Metalloproteinase 9). We selected the top 15 target proteins for further GO (Gene Ontology) and KEGG (Kyoto Encyclopedia of Genes and Genomes) enrichment analysis. The results showed that the genes were mainly enriched in signaling pathways such as PI3K-Akt, TNF, Rap1, MAPK, HIF-1α, Ras, IL-17, VEGF, T cell receptor, and JAK-STAT (Figure 5).

### 3.5 Quantitative proteomics analysis

**FIGURE 6:**
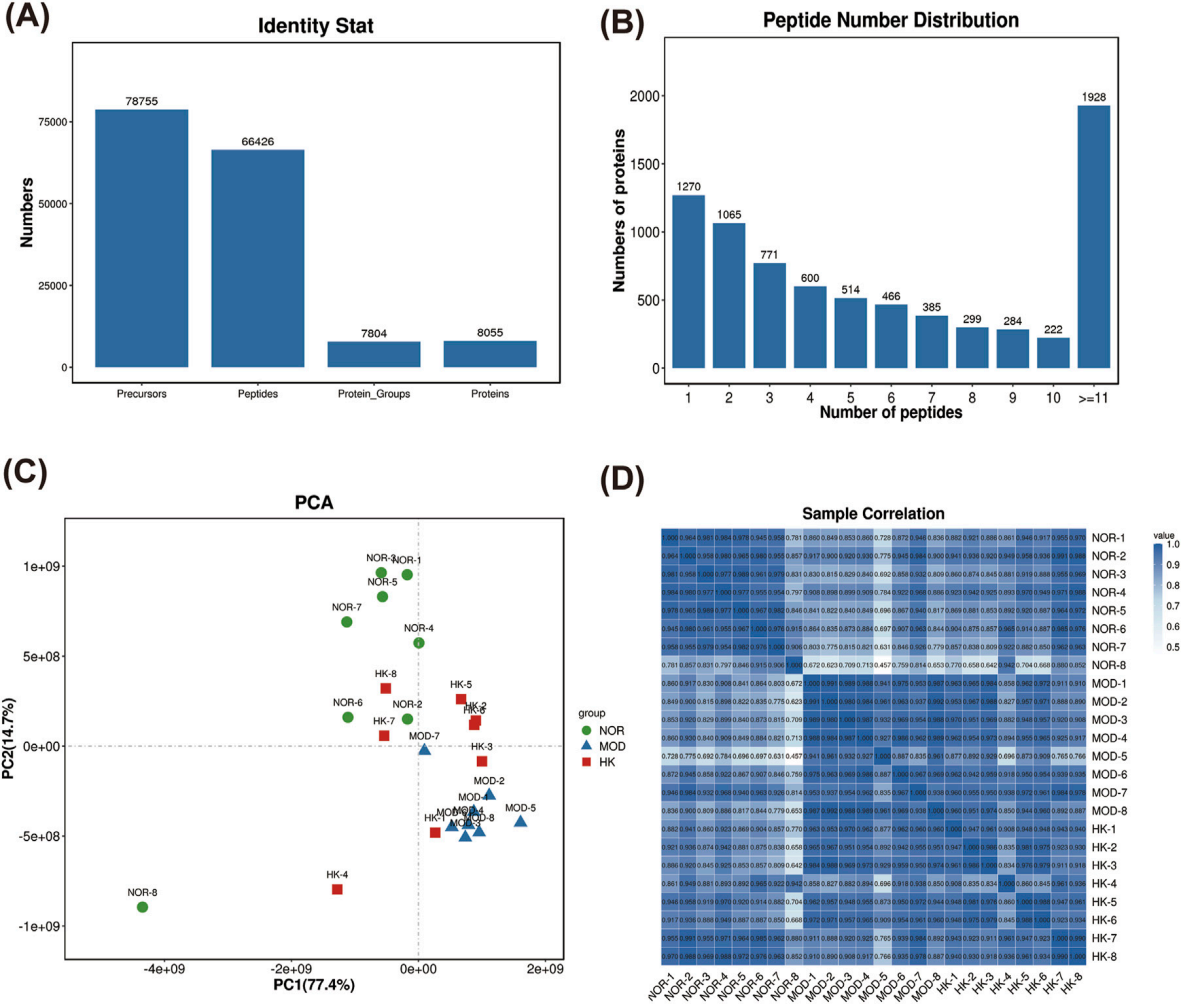
Network pharmacology target analysis. PPI network diagram of the drug composition-target **(A)**. PPI network diagram of disease-target **(B)**. Venn of the CGN and HKC **(C)**. PPI network diagram of the overlapping targets **(D)**.

The protein expression profiles of renal tissues from NOR, MOD, and HK groups were analyzed using the filtering criteria of FDR ≤ 0.01. A total of 66,426 peptide sequences and 8,055 proteins were identified. The highest number of proteins with peptide number more than 11 was 1,928. (Table. 3) (Figures 6A, B).

**TABLE 3:** Protein and peptide identification statistics.

Type	Precursors	Peptides	Protein-groups	Proteins
Numbers	78,755	66,426	7,804	8,055

The sample relationship analysis results showed good similarity within the groups and clear differentiation between the groups, indicating relatively good relationships (Figures 6C, D).

**FIGURE 7:**
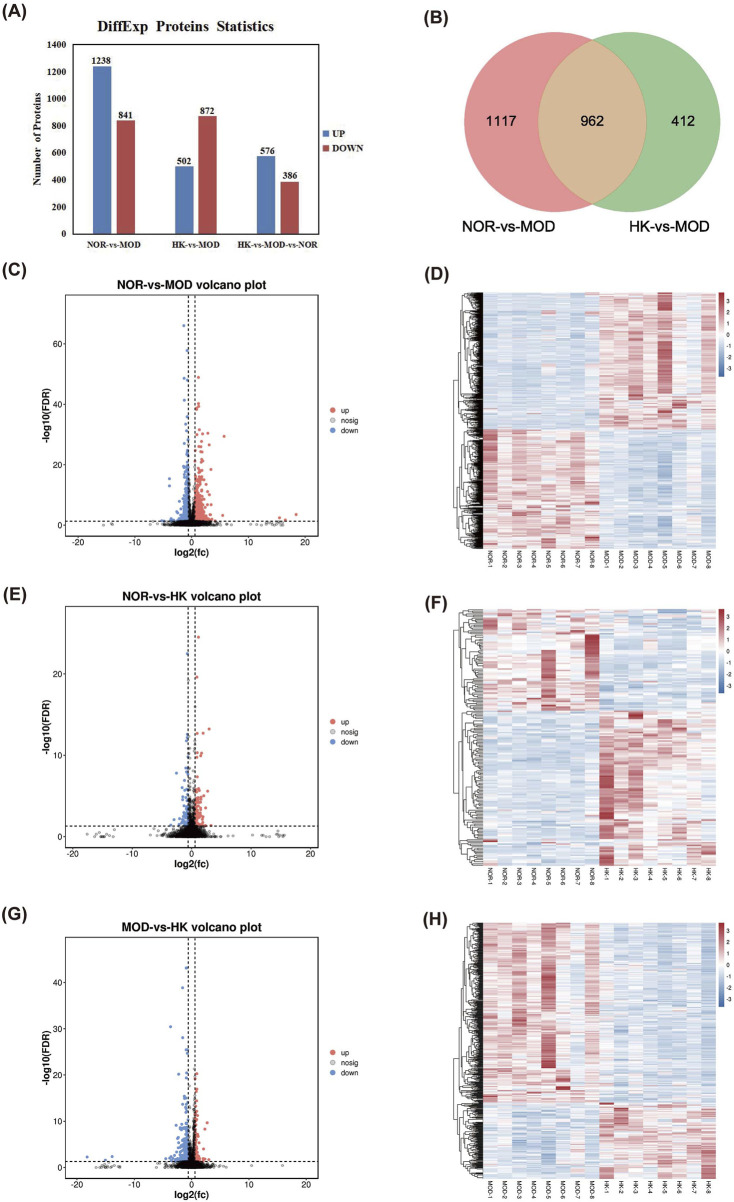
Statistical analysis of differential proteins in NOR, MOD and HK groups. **(A)** Differential protein analysis Veen plots of NOR vs. MOD vs. HK intersecting targets. **(B)** Statistics of differential proteins in the kidney tissues of each group of rats. Volcano plots of NOR vs. MOD **(C, D)**, NOR vs. HK **(E, F)** and MOD vs. HK **(G, H)** with heat maps of differential protein hierarchical clustering analysis.

Using |FC| > 1 and Q value < 0.05 as the criteria for differential protein selection, we statistically analyzed the differentially expressed proteins in the results. We found 2,079 differentially expressed proteins between the MOD and NOR groups, including 1,238 upregulated proteins and 841 downregulated proteins. Additionally, there were 1,374 differentially expressed proteins between the MOD and HK groups, including 502 upregulated proteins and 872 downregulated proteins. A total of 962 differentially expressed proteins were identified among the three groups, consisting of 576 upregulated proteins and 386 downregulated proteins. (Figures 7A, B). The volcano plot and heatmap of hierarchical clustering analysis for the screening results are shown in Figures 7C–H. Detailed information on the significantly different proteins in NOR, MOD, and HK groups after intervention with HKC is provided in Table 4, Figure 7.

**TABLE 4:** List of top-ranked up- and downregulated proteins in terms of multiples of variance.

Gene name	Up-/downregulated protein	Full name of protein	log_2_(fc)
MZB1	Down	Marginal zone B- and B1-cell-specific protein	−4.511464648
A2M	Down	Alpha-2 macroglobulin	−3.678565782
HCLS1	Down	Hematopoietic lineage cell specific protein	−2.994345802
GTF2H2	Down	General transcription factor IIH subunit 2	−2.896066716
PLEKHO2	Down	Pleckstrin homology domain-containing family O member 2	−2.621255328
KNG1	Down	Kininogen-1	−2.263777395
Mup4	Up	Major urinary protein 4	2.706989351
Abca13	Up	ATP-binding cassette subfamily A member 13	2.016518542
NPC2	Up	NPC intracellular cholesterol transporter 2	1.296370706
SCAMP5	Up	Secretory Carrier Membrane Proteins 5	1.13601982
HMGCS2	Up	Hydroxymethylglytaryl-CoA synthetase 2	1.131750831
CHIA	Up	Acidic mammalian chitinase	1.094366751

**FIGURE 8:**
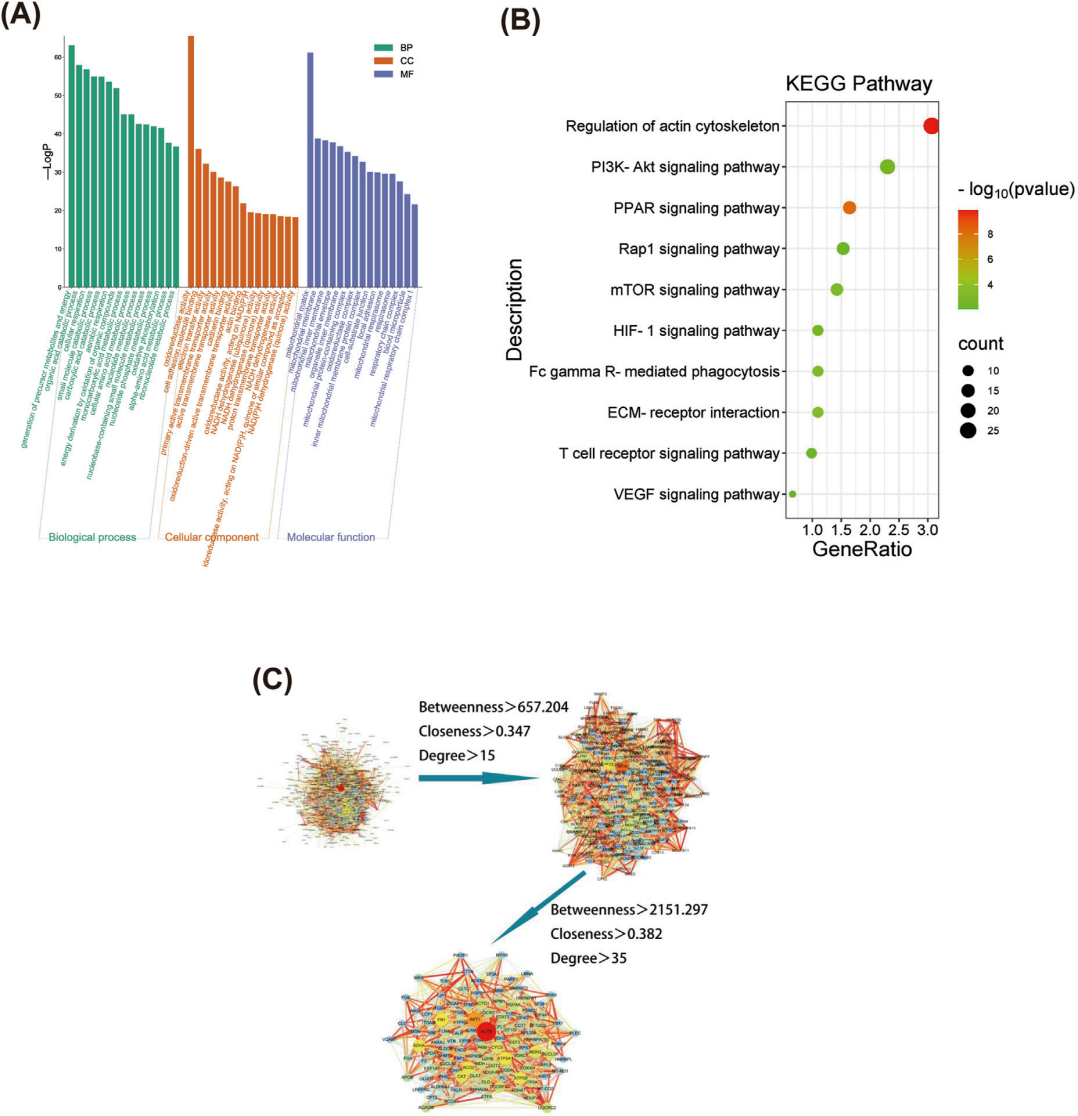
Screening of differential proteins and GO and KEGG enrichment analysis. GO functional enrichment analysis of differential proteins among NOR, MOD and HK groups **(A)**, and the top 20 differential proteins were selected for KEGG enrichment analysis **(B)** Differential protein PPI network construction and core target screening **(C)**.

GO annotation was performed for the 962 differentially expressed proteins selected from the NOR, MOD, and HK groups, resulting in 1,348 biological processes (BP) related to precursor metabolite generation, energy and organic acid metabolic processes, cellular respiration, etc. Additionally, 268 cellular components (CC) were identified, including mitochondrial matrix, mitochondrial membrane, mitochondrial inner membrane, etc. Furthermore, 298 molecular functions (Mbaveng et al., 2020) were associated with oxidoreductase activity, cell adhesion molecule binding, electron transfer activity, and other functions. The top 15 GO analysis results based on P-values were visualized and can be seen in Figure 8.

KEGG enrichment analysis was performed for the 962 differentially expressed proteins selected from the NOR, MOD, and HK groups, resulting in 140 enriched signaling pathways. The top 10 pathways with high relevance were visualized (refer to the figure), indicating that the genes were mainly enriched in signaling pathways such as regulation of actin cytoskeleton, PI3K-Akt, PPAR, Rap1, mTOR, HIF-1α, FcγR-mediated phagocytosis, ECM receptor interaction, T cell receptor, and VEGF (Figure 8).

The interaction networks of differential proteins in NOR, MOD and HK groups were obtained from STRING database. There were 897 node proteins and 9,569 interactions in the interaction network graph, and the PPI network was filtered by CytoNCA plug-in in Cytoscape 3.8.0 software, and the core interaction network of 112 genes was obtained through two filters with the median values of Betweenness, Closeness and Degree as the filtering conditions (Figure 8). There were 112 center nodes and 1,355 edges. ACTB, AKT1, ATP5A1, FN1, SDHA and other proteins are the core nodes in the network, and the detailed node parameters are shown in Table 5.

**TABLE 5:** Top 10 protein topology parameters for protein interaction network degree values.

Gene Symbol	Betweenness	Closeness	Degree
ACTB	90657.76456	0.519721578	178
AKT1	51904.22755	0.496949529	133
FN1	30363.40359	0.456444218	108
ATP5A1	15568.78261	0.452753916	108
ACO2	9119.153372	0.41810546	102
SDHA	8203.124692	0.433268859	102
ATP5B	12434.1115	0.447552448	100
MDH2	8550.283988	0.425451092	97
CAT	31524.29031	0.456444218	95
CYCS	19948.87609	0.457142857	94

### 3.6 Intersected targets between network pharmacology prediction and its validation

**FIGURE 9:**
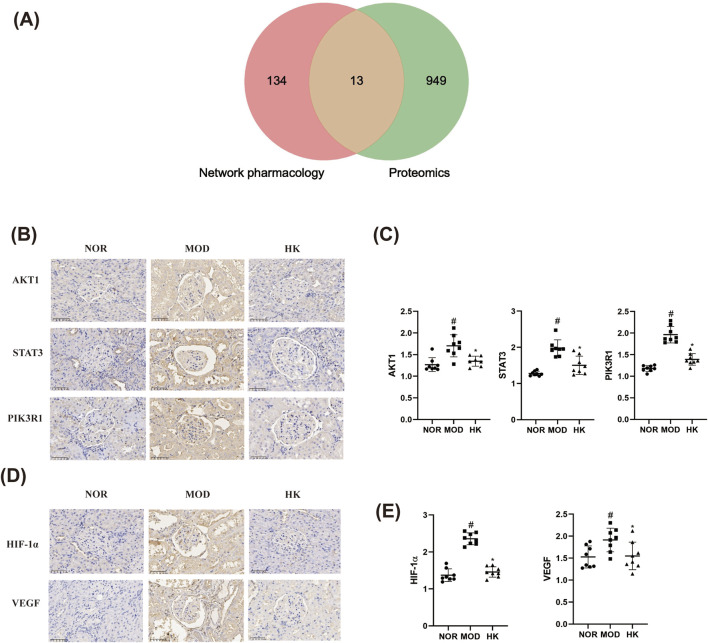
Analysis of the targets and related signaling pathways of CGN treatment by Huangkui capsule (n = 8). **(A)** Veen diagram of core targets of Huangkui capsule based on proteomics and network pharmacology. **(B, C)** Comparison of AKT1, STAT3 and PIK3R1 protein expression levels in the kidney tissues of rats in each group (immunohistochemistry × 400). **(D, E)** Comparison of HIF-1α and VEGF protein expression levels in rat kidney tissues of each group (immunohistochemistry × 400). ^#^p < 0.05 vs. NOR group; *p < 0.05 vs. MOD group.

In this study, we took the intersection of the 962 differentially expressed proteins obtained from proteomics research in the NOR, MOD, and HK groups, and the 147 potential target proteins predicted by network pharmacology for the treatment of CGN with HKC (Figure 9). This analysis led to the identification of 13 common target proteins, including PIK3R1, AKT1, STAT3, F2, PARP1, ICAM1, DPP4, KLKB1, F10, PTPN6, FABP3, SLC37A4, and CASP1. Among them, the most core target proteins, PIK3R1, AKT1, and STAT3, were selected for validation of their protein expression levels using immunohistochemistry, as shown in the Figures 9B, C and Table 6.

**TABLE 6:** Protein expression levels of AKT1, STAT3, and PIK3R1 in kidney tissues of rats in various groups.

Groups	AKT1	STAT3	PIK3R1
NOR	1.268 ± 0.1593	1.277 ± 0.06254	1.180 ± 0.06945
MOD	1.704 ± 0.2559#	1.976 ± 0.2296#	1.963 ± 0.1911#
HK	1.347 ± 0.1154*	1.503 ± 0.2532*	1.390 ± 0.1345*
F	11.94	25.33	66.35
*p*	0.0004	<0.0001	<0.0001

Additionally, 71 pathways were found to be commonly enriched, including HIF-1α, VEGF, PI3K-Akt, mTOR, Rap1, PPAR, FcγR-mediated phagocytosis, T cell receptor, and regulation of actin cytoskeleton, which are related to kidney diseases. The core pathways, HIF-1α and VEGF, were selected for validation of their protein expression levels using immunohistochemistry, as shown in the Figures 9D, E and Table 7.

**TABLE 7:** Protein expression levels of HIF-1α and VEGF in kidney tissues of rats in various groups.

Groups	HIF-1α	VEGF
NOR	1.372 ± 0.1704	1.528 ± 0.2498
MOD	2.356 ± 0.1599#	1.914 ± 0.2695#
HK	1.458 ± 0.1400*	1.550 ± 0.3142*
F	96.01	4.815
*p*	<0.0001	0.0190

## 4 Discussion

**FIGURE 10:**
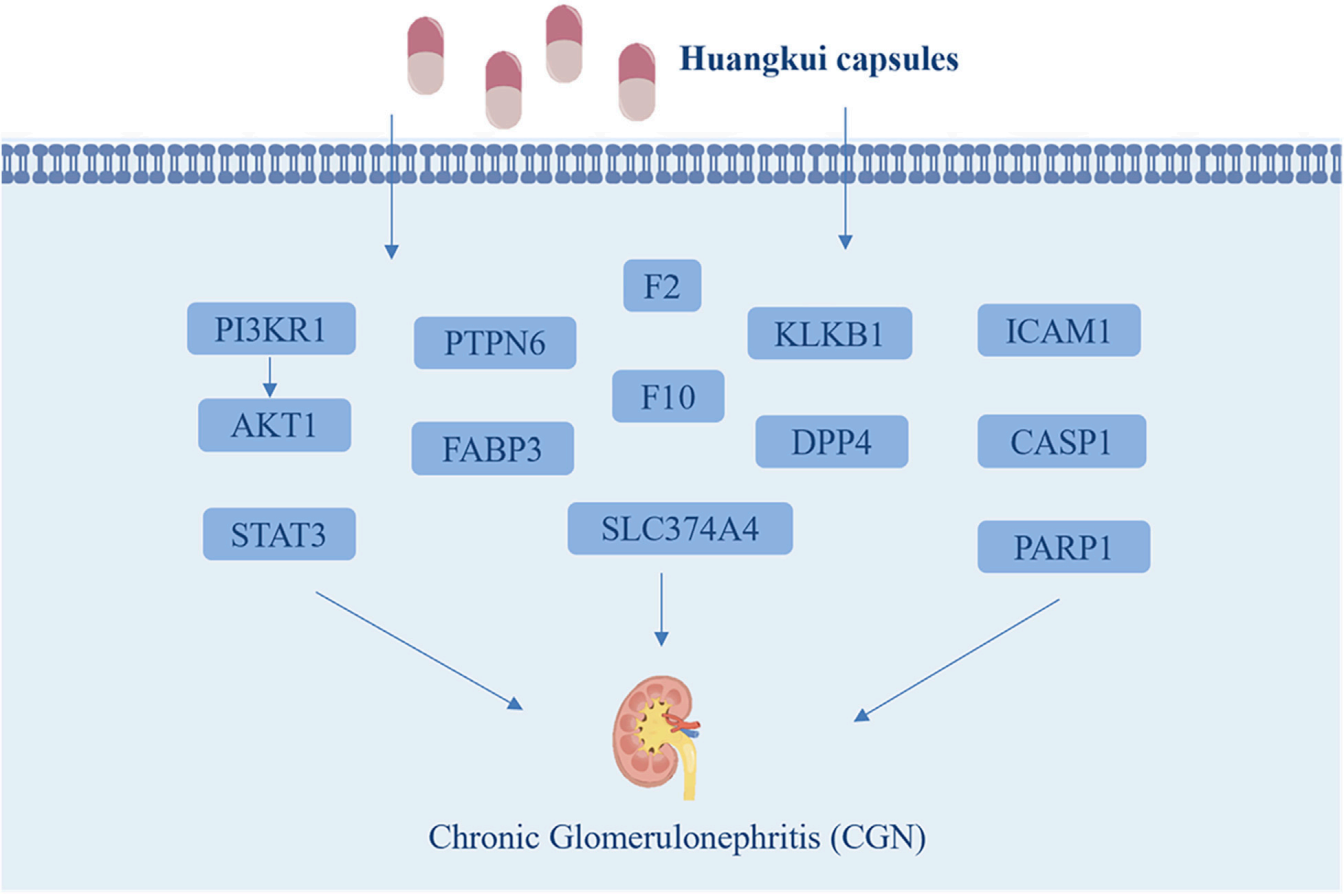
The main possible targets of Huangkui capsule.

This study successfully established a CGN rat model and experimentally verified that HKC significantly alleviates renal injury in CGN rats by reducing kidney-related indicators (24 h UP, PCR, BUN, and Scr) and regulating oxidative stress and inflammation-related proteins (SOD, MDA, IL-6, and TNF-α). Through a combined analysis of network pharmacology and proteomics, 13 key target proteins were identified: STAT3, PIK3R1, AKT1, PARP1, ICAM1, DPP4, KLKB1, F2, F10, PTPN6, FABP3, SLC37A4, and CASP1 (Figure 10). Pathway enrichment analysis revealed that these proteins are primarily involved in HIF-1α, VEGF, PI3K-Akt, mTOR, Rap1, PPAR, FcγR-mediated phagocytosis, T-cell receptor signaling, and actin cytoskeleton regulation. These pathways play critical roles in the pathophysiology of kidney diseases, including renal cell proliferation and apoptosis, fibrosis, inflammation and immune regulation, angiogenesis and vascular permeability, kidney injury, and lipid metabolism. Finally, immunohistochemistry experiments validated the core pathways and key targets, including HIF-1α, VEGF, STAT3, PIK3R1, and AKT1.

It has been clearly demonstrated that the capsule can regulate the kidney through the TGF-β1 and p-p38MAPK signaling pathways, ROSERK1/2-NLRP3 signaling pathway, Akt/mTOR/p70S6K signaling, OMA1-OPA1 axis, SCAP- SREBP2-LDLr signalin (Tu et al., 2013; Ge et al., 2016; Jiang et al., 2019; Li et al., 2019; Wu et al., 2019). Notably, studies have shown that the therapeutic effects of HKC on CGN are primarily attributed to its flavonoid active components. Specifically, quercetin has been demonstrated to regulate the expression of STAT3, PIK3R1, AKT1, CASP1, and ICAM1, thereby inhibiting the release of inflammatory cytokines, reducing oxidative stress damage, and decreasing cell apoptosis. (Ghafouri-Fard et al., 2021; Al-Khayri et al., 2022). Additionally, myricetin and rutin have been shown to modulate various diseases, including diabetes, atherosclerosis, and inflammation-related disorders, through the STAT3, AKT1, and PIK3R1 signaling pathways. (Song et al., 2021). Hyperoside exerts anti-inflammatory and anti-fibrotic effects through the PIK3R1-AKT1-CASP1 axis and mitigates renal tubular injury. (Xu et al., 2022). Therefore, these HKC can ameliorate renal function and attenuate renal tissue injury by targeting multiple pathological pathways involved in the progression of CGN.

Notably, the HIF-1α/VEGF axis plays a pivotal regulatory role in chronic hypoxia and the progression of renal injury (Anjum et al., 2022).Under hypoxic conditions, HIF-1α transcriptionally activates VEGF to promote angiogenesis, however, its aberrant activation may lead to glomerular hyperpermeability, renal tubular vascular dilation, and inflammatory cell infiltration, thereby accelerating renal injury (Gunaratnam and Bonventre, 2009; Izzedine et al., 2010). Concurrently, excessive VEGF-induced vascular leakage may disrupt renal tubulointerstitial homeostasis, further exacerbating fibrotic progression (Alevizakos et al., 2013).

In addition, a large number of studies have shown that AM (Acanthopanax senticosus) has broad therapeutic potential in metabolic diseases, inflammation, and renal protection, with involvement in numerous target pathways. For example, AM can activate peroxisome proliferator-activated receptor (PPAR)-α/γ, alleviate endoplasmic reticulum stress, and play a regulatory role in metabolic diseases (Ge et al., 2016; Kim et al., 2018). In the adriamycin nephropathy rat model, AM can downregulate the expression of TGF-α and TGF-β1, and regulate the p38MAPK signaling pathway as well as the ROS-ERK1/2-NLRP3 inflammasome, thereby inhibiting inflammation and alleviating proteinuria-induced damage (Tu et al., 2013; Li et al., 2019). Additionally, in diabetic animal models, AM can significantly improve renal inflammation by inhibiting the activation of the iRhom2/TACE signaling pathway and downregulating TGF-β1 and p38MAPK (Mao et al., 2015; Liu et al., 2017; Wu et al., 2018). Its main active ingredient, hypericin, can protect podocytes by inhibiting the expression of podocyte heparanase and maintaining the integrity of the glomerular basement membrane in high-fat diet and streptozotocin (STZ)-induced diabetic models (An et al., 2011; Kim et al., 2018).

In immune regulation, AM can alleviate inflammation and exert renal protective effects by inhibiting the NADPH oxidase/ROS/ERK and p38MAPK/AKT signaling pathways (Tu et al., 2013; Zhu et al., 2018; Li et al., 2019). Furthermore, AM can regulate the composition of the intestinal microbiota and influence the Th17/Treg immune balance, further demonstrating its anti-inflammatory and immune regulatory potential (Zhang et al., 2019). These findings suggest that AM has broad therapeutic potential in metabolic diseases, inflammation, and renal protection, but its specific mechanisms still require further research and clinical validation.

In conclusion, based on the above studies, this research hypothesizes that HKC may mediate anti-inflammatory, antioxidant, and renal protective effects through the PI3K-Akt/mTOR-HIF-1α/VEGF signaling pathway crosspoints, providing a theoretical basis for the precise treatment of CGN. However, this study still has certain limitations, such as the lack of multiple protein quantification experiments like Western blot and the failure to explore the mechanisms of specific active monomers of HKC. Therefore, future research will combine *in vivo* and *in vitro* experiments to further validate core targets and signaling pathways, analyze the effects of different flavonoid active components in renal diseases, and ultimately explore their clinical translation potential in patient populations, in order to promote their application in the treatment of CGN.

## 5 Conclusion

Our study reveals that HKC alleviates chronic nephritis by modulating STAT3/PIK3R1/AKT1/HIF-1α/VEGF signaling pathways, reducing oxidative stress, inflammation, and renal injury. While these findings provide mechanistic insights, further validation of HKC’s pharmacological interactions and long-term efficacy is needed. This work demonstrates the utility of proteomics and network pharmacology in elucidating traditional medicine mechanisms, supporting its clinical translation.

## Data Availability

The datasets presented in this study can be found in online repositories. The names of the repository/repositories and accession number(s) can be found below: http://www.proteomexchange.org/, PXD050129.
